# Specificity Study on Concentration of Monoamine Transmitters at Acupoint and Effect of Acupuncture on Its Distribution

**DOI:** 10.1155/2014/704507

**Published:** 2014-12-02

**Authors:** Ting Luo, Yi Guo

**Affiliations:** College of Acupuncture and Moxibustion, Tianjin University of Traditional Chinese Medicine, Tianjin 300193, China

## Abstract

To investigate the distribution of monoamine transmitters at acupoints and effect of acupuncture on it. Take this experiment by means of microdialysis. Twenty rabbits were randomly assigned to two groups (Group A: acupuncture Guanyuan (RN4), Group B: acupuncture nonacupoint which is besides Guanyuan (RN4) 1 cm). Before and after acupuncture was taken, tissue fluids both at Zhongwan (RN12) which is on the same meridian as Guanyuan and at a nonacupoint 1 cm away from Zhongwan were collected through microdialysis, respectively. The collected samples were analyzed to determine concentrations of monoamine transmitters. Epinephrine and 5-HT were detected. An unknown substance was found. Its concentration at acupoint was significantly higher than that at nonacupoint and decreased after acupuncture. Its significant specificity at acupoints suggests that it may play an important role in meridian's activity.

## 1. Introduction

Researchers have carried on research from the perspective of biochemistry of the meridians for years. It has proved that ions [1, 2], oxygen metabolism [3], nitric oxide (NO) [4], adenosine [5], and so forth are closely associated with the meridian activities. Some scholars believe that the meridians are the sensitive lines of sympathetic nerve [6]. The transfer process of acupuncture signal depends on a kind of low weight molecular substances moving along the meridian [7]. In addition, it has suggested that acupuncture can stimulate the release of monoamine transmitters in the incision liquid extract from meridian [8]. However, the skin incision as a mechanical stimulation can also cause skin to release certain biochemical substances, and it cannot distinguish whether such substances only release from the meridians. In consideration of this, we carried out the study by means of microdialysis attempted to reduce this interference to a minimum.

## 2. Subjects and Methods

### 2.1. Experimental Animals

20 healthy rabbits (9 female, 11 male; weighing 2–2.7 kg; provided by the Animal Center of Tianjin University of Traditional Chinese Medicine) were randomly divided into two groups, each group has 10 rabbits. Group A received manual needle acupuncture at Guanyuan (RN4) acupoint and group B received manual needle acupuncture at nonacupoint besides Guanyuan (RN4) 1 cm.

### 2.2. Main Instrument

Consider the following: microperistaltic pump (England Watson-Marlow, 205 U); Shimadzu LC-6A HPLC; CTO-6A column oven; L-ECD-6A electrochemical detector (Sensitivity: 1 AUF); C18 column (10 *μ*, 250 mm × 4.6 mm, Tianjin dimensional chromatographic company).

### 2.3. Experimental Methods

#### 2.3.1. Preparation of the Microdialysis Probe (See Figure 1, Illustration of the Microdiasis Probe (Concentric Type [9]))

A 6th stainless steel needle is obliquely inserted into polyethylene plastic pipe, then a capillary plastic tube, whose outside diameter has been stretched in the range of 160–180 *μ*m, is inserted in the stainless steel needle with 0.3 cm exposed outside. We advanced the stunt end of steel needle into the polyethylene plastic pipe and the tip of the needle exposed for 2 cm. A dialysis tube, molecular weight cut-off is 50 KD, 1 cm long, then is carefully advanced from the stainless steel needles tip, and exposed about 2.5 mm than the needle. Sealed the end of the dialysis tube, the junction between stainless steel needle and dialysis tube, needle and polyethylene plastic pipe, polyethylene plastic pipe, and plastic capillary tube, placed for some time to be solidified.

#### 2.3.2. Probe Detection and Calibrations

In order to test the stability of the probe, a series of experiments have been made. It has been found that single probe, with increasing frequency of use and its recovery, is decremented. And the recovery of several probes has no difference. So the repeated use of single probe is avoided in the experiment.

To further test the stability of the probe recovery, we measured eight-dialysis-probe recovery of monoamine neurotransmitters stability. Methods: eight probes were immersed in a concentration of monoamine neurotransmitters 200 ng/mL standard solution 100 *μ*L (norepinephrine (NE), epinephrine (E), 3,4-dihydroxybenzoic acid (DHBA), dopamine (DA), and 5-hydroxytryptamine (5-HT) join in 0.1 N HCl and formulate the standard solution. The standard solution is provided by Sigma), perfused for 30 min, perfusion solution is Ringer's solution, pump speed is 2 *μ*L/min, and temperature is 20°C. And the probe is placed in a stirred homogeneous solution [10]. Collected dialysis solutions are analyzed by HPLC-ECD. For results see Table 1.

#### 2.3.3. Tissue Fluid Collection

Fix the rabbit on rabbit stationary, cut off part of the rabbit hair about 3 cm in diameter at the monitoring point (Zhongwan (RN12) and 1 cm besides Zhongwan (RN12)), skin disinfection with iodine-tincture 2% and alcohol 75%. Two microdialysis probe introducers prepared in advance (75% alcohol soaked), respectively, were inserted in Zhongwan (RN12) and 1 cm besides Zhongwan (RN12) with a 45-degree angle, depth of 2 mm. Then two dialysis probes, connected with microperistaltic pump, were carefully inserted into the introducer. Perfusion fluid used Ringer solution having a composition of Na^+^ 151.7 mmol/L, K^+^ 2.69 mmol/L, Ca^2+^ 1.1 mmol/L, Cl^−^ 152.3 mmol/L, pH: 7.4. Perfusion rate is 2 *μ*L/min and was stabilized for 1 h. Collected tissue fluid at two monitor points in a dark place with 0.2 mL centrifuge tubes for 30 min. After this collection, collected another samples also in a dark place with 0.2 mL centrifuge tubes for 30 min. While, at the same time, acupuncture Guanyuan (RN4) and non-acupoint 1 cm away from Guanyuan in twirling and lifting-thrusting method for 5 min with 1 cun acupuncture needles, and then retain needles until the collection is finished. Collected samples were put in liquid nitrogen storage tank until being detected.

#### 2.3.4. Detection Method

A total of 80 samples were collected and detected by HPLC-ECD. The chromatographic conditions were as follows: C18 column (4.6 mm × 250 mm, 10 *μ*) phosphate buffer (pH 3.2) as mobile phase at a flow rate of 0.8 mL/min. The standard solution is prepared by adding 0.1 N HCl in Norepinephrine (NE), epinephrine (E), 3,4-dihydroxybenzoic acid (DHBA), dopamine (DA) and 5-hydroxytryptamine (5-HT) which are produced by Sigma company.

### 2.4. Statistical Analysis

The data were analyzed using SPSS 11.0 software. Testing was performed with Paired-samples* T*-test.

## 3. Results

**Figure 2:**
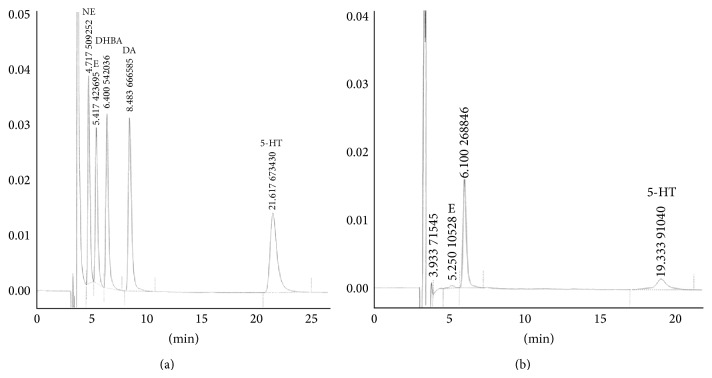
Chromatogram of monoamine transmitter at rabbit's acupoint (a) standard solution (b) dialysate from rabbit's acupoint.

Epinephrine (E) and 5-HT are detected in a few samples with low concentrations. In addition to E and 5-HT (see Figure 2), most samples have only one peak, and retention time is between 5.8 and 6.1 min. It cannot be concluded what it is. For standards spectra see Figure 2.

Through study on the spectrum of the high peak of the unknown substance, we find that (1) there was a very significant difference (*P* < 0.01) on area of the unknown substance between Zhongwan (RN12) and nonacupoint besides Zhongwan (RN12) 1 cm (Table 2). (2) The area of the unknown substance reduced after acupuncture Guanyuan (RN4) (Table 3). It has a significant difference (*P* < 0.05). (3) Acupuncture Guanyuan (RN4) also can make area reduced at nonacupoint, but there was no significant difference (*P* > 0.05) (Table 4). (4) Acupuncture nonacupoint makes area increased at the point of Zhongwan (RN12), but there was no significant difference (*P* > 0.05) (Table 5). (5) The area of nonacupoint besides Zhongwan (RN12) 1 cm increased after acupuncture nonacupoint away from GuanYuan (RN4) 1 cm, and there was no significant difference (*P* > 0.05) (Table 6).

## 4. Discussion

### 4.1. Experimental Results Analysis

In this study, it has observed that monoamine neurotransmitter levels in dialysate of acupoints and nonacupoints is very low. It is because of two reasons: firstly because the concentration itself is very low at acupoints and nonacupoints and secondly it is due to the microdialysis probes' low recovery especially that of 5-HT, its recovery is less than 3% (see Table 1 monoamine neurotransmitters recovery testing).

Nevertheless, E and 5-HT had been tested in the experiment. It is generally believed that E only exists in the outer periphery of the adrenal medulla cells, so some scholars think that this will certainly have meaningful impact on the study of sympathetic substances on the skin [11]. It has detected very low concentrations of 5-HT at points. It is seen from the detected 27 cases in the sample that there are no differences at 5-HT concentration between acupoint and nonpoint. Regardless of acupoint or nonpoint, concentration of 5-HT in the Guanyuan acupuncturing group is more than the nonacupoint acupuncture group. This trend should be further verified in further experiment by improving recovery of microdialysis probe.

The study also found an unknown substance in the spectrum. It has suggested that (a) the distribution of the unknown substance has significant specificity and the concentration at acupoints is greater than that of nonacupoints, (b) acupuncturing acupoints can make its concentration of acupoints on the same meridian reduced, (c) while acupuncturing acupoints, it has little effect or no effect on its concentration of nonacupoints, (d) it has little effect or no effect on acupoints or nonacupoints, while it is stick nonacupoint. It has also suggested that the unknown substance has a specificity at the meridian and plays a role in the meridian activities.

To learn more about the structure of this unknown matter, we used liquid chromatography-mass spectrometry to analyze the dialysis samples. However, because of the high sensitivity of inorganic ions in dialysate solution, it has overshadowed the spectra of the unknown substance. The analysis results are not ideal. And due to the restriction of the sample size, it does not seek an effective method for separating inorganic salts. Therefore, the analysis of unknown structure did not succeed.

In the study of incision fluid along meridian, Liu has detected high concentration of noradrenaline (NA or NE) in some samples, and as for adrenaline (E), he thought it is due to the lack of experimental injection volume it did not detect [11]. Comparing to spectrum of the incision fluid, it is very similar to our experimental spectrum. Liu believes that the peaks that retention time is 7.56 min and 26.63 min are mainly intermediate metabolites of catecholamine. It provides some ideas for our future research.

### 4.2. Function of Monoamine Neurotransmitters In Vivo and Relationship with Meridian Biochemical Studies

Studies have shown that skin of higher animal contains norepinephrine and epinephrine, but no dopamine [12]. Norepinephrine in skin is released primarily by sympathetic nerve endings, while the adrenaline is released by pheochromocytoma scattered in the skin. Experiments show that acupuncture can cause skin to release NA, and the release follows the meridians. NA acts on *α* receptor, and while *α* receptor is blocked, it can cause the block of propagated sensation along meridian and acupuncture effect. The agonists of *α* receptor injected into skin can produce a strong acupuncture effect. Obviously, catecholamines in skin mediate the transduction of meridians and effects of acupuncture. It can infer that the release of NA in meridians by acupuncturing is caused by sympathetic reflex, and neurotransmitters directly diffuse along meridians [13]. Therefore, some scholars believe that the substance of the meridian is sympathetic and sensitive lines in vivo [6]. And by using macroautoradiography method it shows a series of sympathetic substance lines [7]. The skin NE and 3H-NE concentration release in acupoints was significantly higher than those in nonacupoints and nonmeridian controls. Consistently, enhanced 3H-NE synthesis/release in acupoints/meridians is facilitated by the presence of an exogenous NO donor and inhibited by an inhibitor of NO synthesis [14]. And studies show that electroacupuncture stimulation induces a significant release of NO and cGMP in the acupuncture acupoint but not in the nonmeridian area [15]. 5-HT is a kind of pain producing substance in peripheral, and some scholars have found that acupuncture can reduce 5-HT and DA levels at inflammation area [16]. In conclusion, monoamine neurotransmitters have a certain relationship with acupuncture in peripheral mechanism of acupuncture.

## Figures and Tables

**Figure 1:**
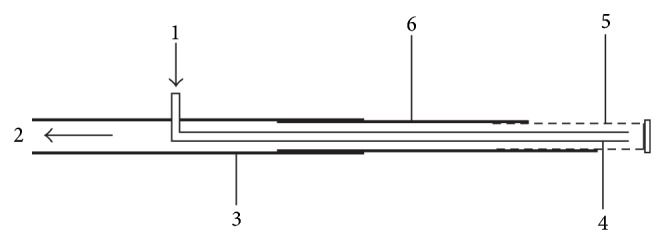
Illustration of the microdialysis probe (concentric type). The parts are (1) inlet tubing, (2) outlet tubing, (3) polyethylene plastic pipe, (4) 6th stainless steel needle, (5) dialysis tube, and (6) capillary tube.

**Table 1:** Monoamine neurotransmitter recovery testing.

*N* = 8	NE	E	DHBA	DA	5-HT
Recovery % (*X* ± SD)	6.02 ± 1.00	7.23 ± 1.42	68.16 ± 26.72	5.49 ± 1.07	2.78 ± 1.13

**Table 2:** Comparison of the unknown peak area between acupoint and nonacupoint.

	Zhongwan (RN12) (*n* = 20)	Nonacupoint (*n* = 20)	Difference	*T*
Area	333580.95 ± 174941.38	204789.60 ± 91674.55	128791.35 ± 179245.84^*^	3.213

^*^ *P* < 0.01.

**Table 3:** Changes of unknown peak area at acupoint after acupuncturing the acupoint.

	Zhongwan (RN12)			
	Before acupuncture	After acupuncture	Difference	*T*
Area	342006.30 ± 169110.99	237584.60 ± 88476.09	104421.70 ± 129672.31^*^	2.546

^*^ *P* < 0.05.

**Table 4:** Changes of unknown peak area at nonacupoint after acupuncturing the acupoint.

	Away from Zhongwan (RN12) 1 cm (*n* = 10)			
	Before acupuncture	After acupuncture	Difference	*T*
Area	221634.70 ± 100452.24	204506.10 ± 197752.34	17128.6 ± 202874.19^*^	0.267

^*^ *P* > 0.05.

**Table 5:** Changes of unknown peak area at acupoint after acupuncturing the nonacupoint.

	Zhongwan (RN12) (*n* = 10)			
	Before acupuncture	After acupuncture	Difference	*T*
Area	330670.60 ± 186440.01	360456.50 ± 242999.06	−29785.9 ± 313007.28^*^	−0.301

^*^ *P* > 0.05.

**Table 6:** Changes of unknown peak area at nonacupoint after acupuncturing the nonacupoint.

	Away from Zhongwan (RN12) 1 cm (*n* = 10)			
	Before acupuncture	After acupuncture	Difference	*T*
Area	187944.50 ± 83791.55	200214.30 ± 153597.45	−12269.8 ± 199318.27^*^	−0.195

^*^ *P* > 0.05.
